# Supplementary material for: The Adsorption Behavior of Gas Molecules on Co/N Co–Doped Graphene
Source: Molecules. 2021 Dec 20;26(24):7700. doi: 10.3390/molecules26247700 (PMC8704436; doi:10.3390/molecules26247700)
Supplement: Supplementary file 1 [file molecules-26-07700-s001.zip › molecules-1498056-supplementary.pdf]

Table S1. The distance between gas molecules and Co atom (dCo-gas/Å), the charge transferred to the gas molecules (ΔQgas/e), adsorption energies (Ead/eV), and the total magnetic moment of the systems (M/μB), after gas adsorption.

Gas	$d_{Co\text{-}gas}$ (Å)	ΔQ_{gas} (e)	E_{ad} (eV)	M (μ_B)
C_2H_2	1.92	-0.38	-1.95	2.00
CO	1.76	-0.31	-2.27	2.00
NO_2	1.92	-0.70	-3.32	1.00
SO_2	1.93	-0.71	-1.85	0.14

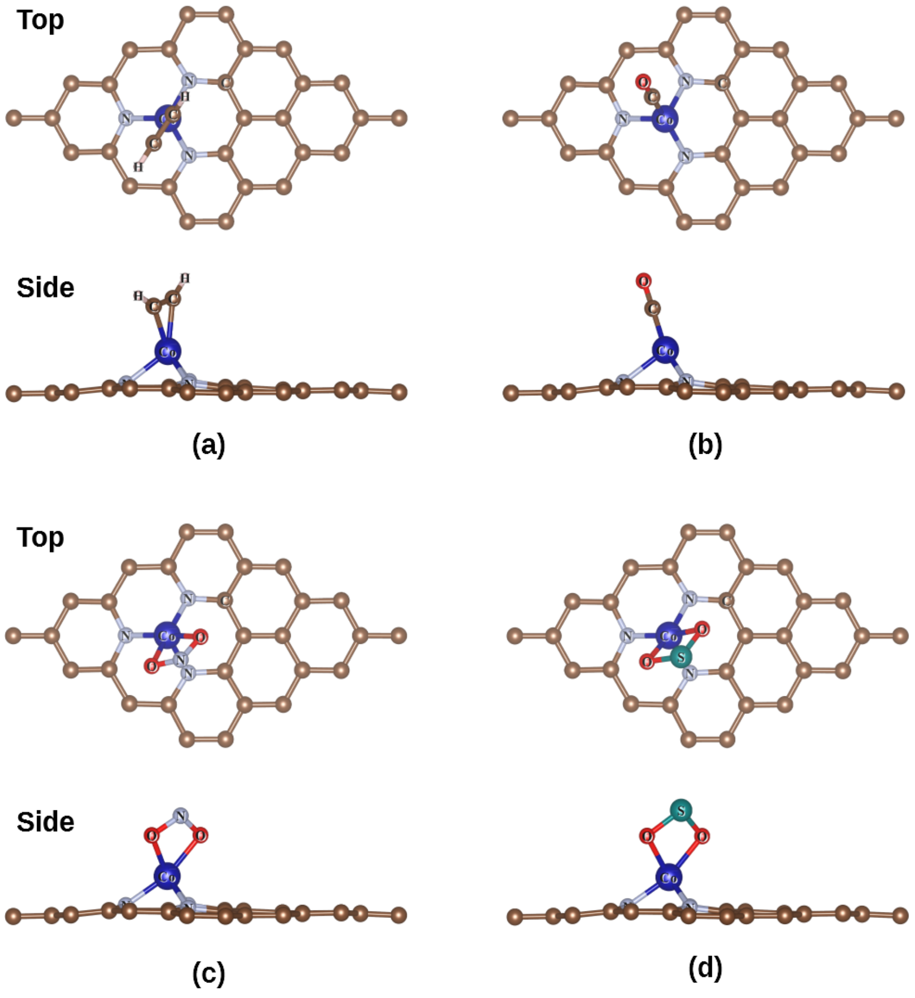

Figure S1. The top and side view of configurations of Co/N_3-gra with (a) C_2H_2, (b) CO, (c) NO_2, and (d) SO_2 adsorbed on it.

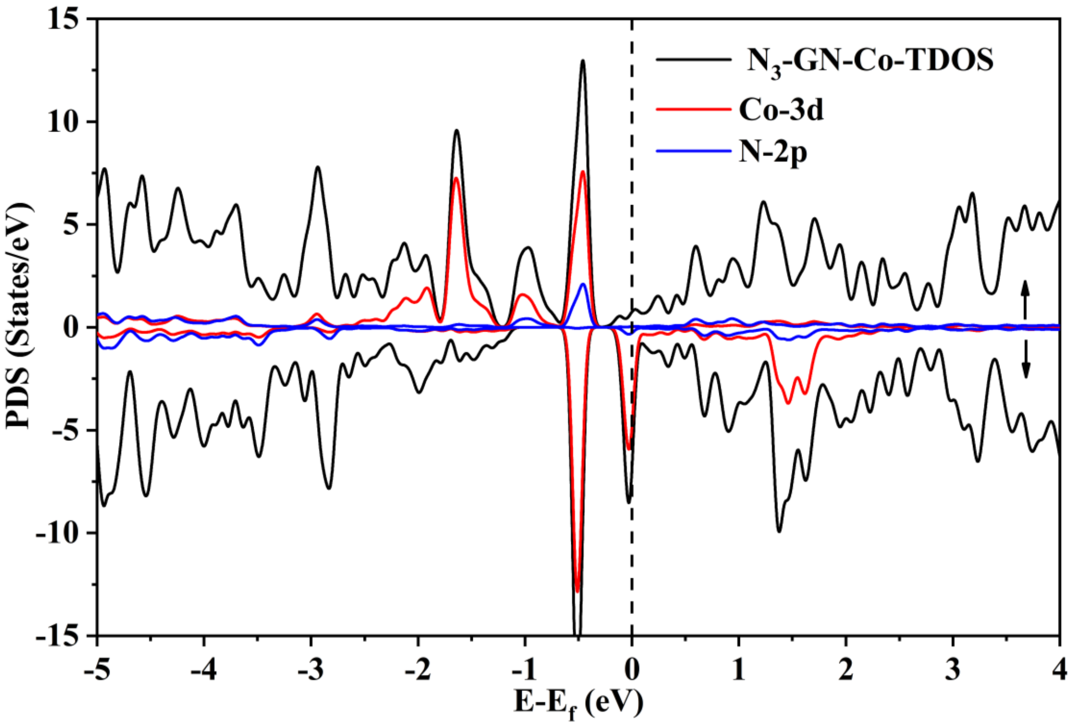

Figure S2. The density of states of Co/N_3-gra, with the up (down) spin denoted as ↑ (↓). The dotted line indicates the Fermi level.
